# Application of Hydrogen Peroxide Encapsulated in Silica Xerogels to Oxidation Reactions

**DOI:** 10.3390/molecules17078068

**Published:** 2012-07-04

**Authors:** Szczepan Bednarz, Barbara Ryś, Dariusz Bogdał

**Affiliations:** Chair of Biotechnology and Renewable Materials, Cracow University of Technology, Warszawska 24, 31-155 Krakow, Poland; Email: rysq86@o2.pl (B.R.); pcbogdal@cyf-kr.edu.pl (D.B.)

**Keywords:** oxidation, epoxidation, microwave irradiation, silica xerogel, hydrogen peroxide, sol-gel, fluoroalcohols, urea-hydrogen peroxide

## Abstract

Hydrogen peroxide was encapsulated into a silica xerogel matrix by the sol-gel technique. The composite was tested as an oxidizing agent both under conventional and microwave conditions in a few model reactions: Noyori’s method of octanal and 2-octanol oxidation and cycloctene epoxidation in a 1,1,1-trifluoroethanol/Na_2_WO_4_ system. The results were compared with yields obtained for reactions with 30% H_2_O_2_ and urea-hydrogen peroxide (UHP) as oxidizing agents. It was found that the composite has activity similar to 30% H_2_O_2_ and has a several advantages over UHP such as the fact that silica and H_2_O are the only products of the composite decomposition or no contamination by urea or its derivatives occurs; the xerogel is easier to heated by microwave irradiation than UHP and could be used as both an oxidizing agent and as solid support for microwave assisted solvent-free oxidations.

## 1. Introduction

One of the methods for the preparation of porous materials is sol-gel processing [1]. The method is characterized by the formation of stable colloidal solutions (sol) in the first step followed by anisotropic condensation of colloidal particles producing a polymeric network with an entrapped solution. After washing and removal of the solvent xero- or aerogels are formed depending on the drying mode. The sol-gel technique is employed for the production of porous solids, such as silica and alumina, widely used as catalyst supports. Various metal substituted silica matrices were synthesized and catalytic activity in oxidation reactions was demonstrated. For example titania-silica xerogels and aerogels were used as a catalyst for oxidation reactions with H_2_O_2_ or *tert*-butyl hydroperoxide as oxidants [2]. The oxidation of some arenes with alkyl side groups by H_2_O_2_ with tungstoboric acid supported on SiO_2_ xerogels was reported [3]. Additionally, a xerogel composite of V_2_O_5_ was evaluated as a catalyst for the epoxidation of styrene and cyclooctene using iodosylbenzene, H_2_O_2_ and *m*-chloro-perbenzoic acid as oxidants [4]. Metallosilicate compounds, prepared by dispersion of metal oxide (TiO_2_, MoO_3_ or WO_3_) in amorphous silica, were catalytically active in the oxidation of alkenes and alcohols with 30% H_2_O_2_ solution [5].

Sol-gel processed porous silica glasses also have considerable potential as carriers for controlled drug and temperature-sensitive molecule release [6]. Recently, a sol-gel method was successfully applied for incorporation of H_2_O_2_ into a silica xerogel matrix to obtain a new antimicrobial composite for medical applications [7]. It was demonstrated that the xerogel was biologically active to nearly the same extent as H_2_O_2_ solutions [7]. The composite containing up to 68% of H_2_O_2_ was stable because of the formation of strong hydrogen bonds between siloxane oxygens and H_2_O_2_ [7,8]. The studies have shown that H_2_O_2_ was quickly and fully released from the composite into water [7].

To the best of our knowledge, silica xerogel-H_2_O_2_ composites have not been used in organic synthesis as an oxidant. This paper demonstrates a successful application of these xerogels as a H_2_O_2_ source for the oxidation of aldehydes, secondary alcohols and epoxidation of cycloolefins, under both conventional and microwave conditions.

## 2. Results and Discussion

**Figure 1:**
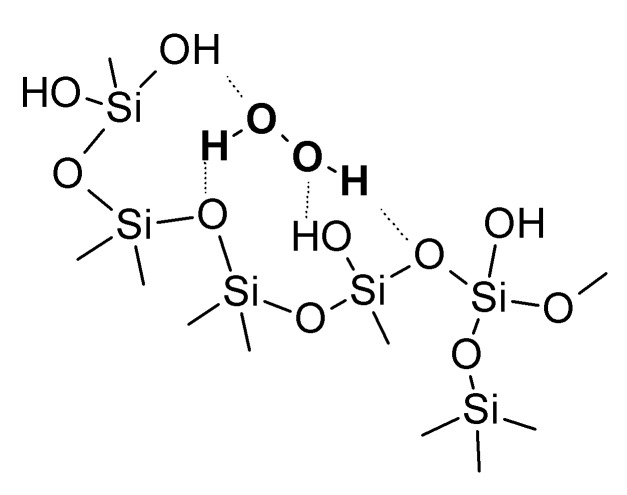
A simplified structure of silica xerogel-hydrogen peroxide composite [7,8].

**Figure 2:**
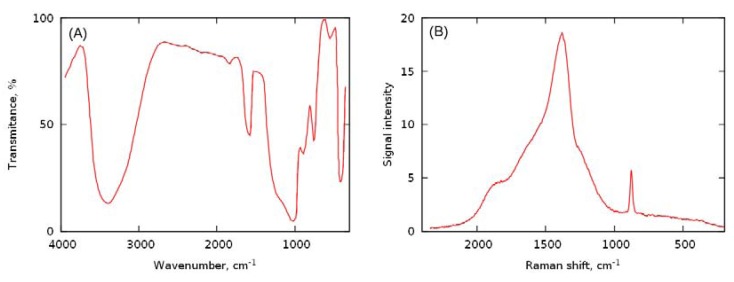
Spectroscopic characterization of silica xerogel-hydrogen peroxide composite: (**A**) FTIR spectrum, (**B**) Raman spectrum.

Drying of a mixture of H_2_O_2_ in silicic acid sol results in the evaporation of H_2_O and partially of H_2_O_2_. Simultaneously, silicic acid undergoes condensation and polymerization (crosslinking) followed by an aggregation. Further drying caused the transformation of the silica gel into a silica xerogel and entrapment of both H_2_O and H_2_O_2_ inside the silica network (Figure 1). The IR spectrum (Figure 2A) of the composite is similar to described in the literature [8] and shows characteristic peaks of hydrated silica (broad band 3700–3000 cm^−1^ O–H stretching frequencies, 1630 cm^−1^ molecular bending vibrations of water, 1400–1000 cm^−1^ deformation vibrations of the hydroxyl of Si-OH) and H_2_O_2_ (1350 cm^−1^ H–O–O bending band). Additionally, the Raman spectrum [Figure 2B] shows a peak at 876 cm^−1^ clearly indicating presence of the peroxide. The amount of encapsulated H_2_O_2_ and the mass ratio of H_2_O_2_/H_2_O depend on drying time and temperature, which was monitored by weighing the composite during drying [7]. The determined average composition of the obtained composites was 40% H_2_O_2_, 43% H_2_O and 17% SiO_2_. It was found that loading efficiency of H_2_O_2_ was c.a. 50%, thus half of the initial amount of H_2_O_2_ in a sol was encapsulated in the final product and the rest was lost during drying of the sol.

**Scheme 1:**
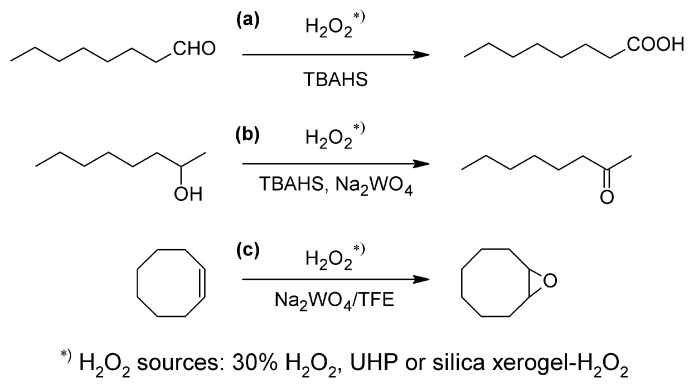
The model reactions: oxidation of octanal (**a**), 2-octanol (**b**) and epoxidation of cyclooctene (**c**).

To investigate the activity of H_2_O_2_ encapsulated in silica xerogel, some model reactions were examined such as Noyori’s procedures for solvent-free aldehyde [9] and alcohol [10,11] oxidation and epoxidation of cyclic olefins in fluorinated alcohol as a solvent [12] (Scheme 1). For comparison the reactions were also carried out with 30% H_2_O_2_ solution and urea-hydrogen peroxide adduct (UHP) as oxidant. Additionally, microwave irradiation was employed to shorten the reaction times and to increase their yields.

The oxidation of octanal (Table 1) both by the 30% of H_2_O_2_ and H_2_O_2_ encapsulated in the xerogel gave very good yield of the acid (>80%). Application of UHP resulted in lower conversion of octanal (49%) and, additionally, under microwave condition side reactions took place-most probably condensation of urea with the aldehyde [14]. For that reason, in spite of high conversion, the reaction product was not detected by GC.

**Table 1:** Effect of a source of H_2_O_2_ on the oxidation of octanal under conventional and microwaves conditions.

H_2_O_2_ source	Yield of octanoic acid, %	
	Conventional conditions,	Microwave irradiation,
	90 °C, 120 min	90-95 °C, 45 min
30% H_2_O_2_	86	81
UHP	49	0 ^a^
The xerogel	83	73

^a^ Several undefined by-products were detected by GC and the product (octanoic acid) was not affirmed.

2-Octanol was successfully oxidized in nearly quantitative yield both by the composite and 30% aqueous H_2_O_2_ (Table 2). The application of UHP as an oxidant lowers the yield of the reaction to 69%. Moreover, when the reaction temperature was decreased to 70 °C, UHP exhibited low reactivity manifested only 6% yield of 2-octanone with comparison to 64% and 56% for aqueous H_2_O_2_ and the xerogel, respectively. Oxidation under microwave conditions gave very good results only when 30% H_2_O_2_ was applied.

**Table 2:** Effect of the source of H_2_O_2_ on the oxidation of 2-octanol under conventional and microwaves conditions.

H_2_O_2_ source	Yield of 2-octanone, %	
	Conventional conditions, 80 °C, 30 min	Microwave irradiation, 80-90 °C, 15 min
30% H_2_O_2_	92 (64 ^a^)	99
UHP	69 (6 ^a^)	<1
The xerogel	98 (56 ^a^)	46

^a^ Reactions carried out at 70 °C (below melting point of UHP).

In further studies, the yield of cyclooctene oxide was highest for reaction with UHP (94%) as an oxidant, while slightly lower yield (88%) was observed for reactions with 30% H_2_O_2_. However, in the presence of the xerogel, 58% conversion of the olefine was observed. Elongation of the reaction time or increasing the amount of the xerogel improved the yield.

**Table 3:** Effect of the source of H_2_O_2_ on the epoxidation of cyclooctene under conventional and microwaves conditions.

H_2_O_2_ source	Yield of cyclooctene oxide, %	
	Conventional conditions, 3 h 60 °C	Microwave irradiation, 1 h 60–70 °C
30% H_2_O_2_	88	90
UHP	94	95
The xerogel	58 (69 ^a^, 74 ^b^)	71

^a^ Reaction time was 4 h. ^b^ Highest amount of the xerogel was used (equal 14 mmol H_2_O_2_).

Silica xerogel–hydrogen peroxide composites stabilized by addition of small amounts of H_3_PO_4_ can be stored at 3 °C for 2 months without significant loss of H_2_O_2_ [7]. However, we have used unstabilized composites. We have found that after 2 weeks of storing at 5 °C, the composite loses about a half amount of H_2_O_2_ probably because slow decomposition of H_2_O_2_ catalyzed by heavy metals impurities presented in the silica matrix (probably, traces of Fe^2+^ from “water glass”). Relative instability of the composite could be a reason of its activity in oxidation reactions. In contrast, UHP is a stable H_2_O_2_ complex and such a high stability could be considered, to be a drawback to its potential chemical reactivity. An oxidation yield might by higher if dissociation of the adduct, *i.e.*, dissolving or melting (m.p. 82 °C [15]) took place. UHP/hexafluoro-2-propanol (HFIP) system was investigated in epoxidation of olefins, and it was concluded that the fluoroalcohol has the unique ability to combine the two requirements: dissociation (dissolving) of the UHP adduct and activation of H_2_O_2_ [16]. We have observed similar effect of TFE on the epoxidation yield of cyclooctene with UHP as an oxidant (Table 3). The temperature effect on UHP and the silica xerogel–H_2_O_2_ composite activity was investigated in 2-octanol oxidation (Table 2). At the temperature 70 °C (below melting point of UHP) a conversion of the alcohol was very low and was significant increased when the temperature risen to 80 °C. In opposition, oxidation with 30% H_2_O_2_ and also with the composite did not show such high yield–temperature dependency.

We have found that silica xerogel–H_2_O_2_ composite is more suitable oxidant to microwave assisted reactions than UHP. The composite is easily heated by microwave irradiation probably because they contain c.a. 80% of polar components: H_2_O_2_ and H_2_O. These molecules are relatively weakly bonded to silica matrix and can undergo reorientation in microwave field, resulting in heat generation. Silica matrix is almost transparent for microwave irradiation (ε” < 0.001 2.5 GHz [17]) and does not heated directly by the radiation. For this reason, undesired superheating effect of the xerogel under microwave condition is much less probably than for solids strongly interacted with microwaves such as chromium dioxide (Magtrieve™) [18]. UHP is a crystalline complex formed by hydrogen bonding between urea and H_2_O_2_ [15], thus the molecules are locked together in a crystal structure. From that reason solid UHP weakly interacts with microwave irradiation in contrast to the melted one, which strongly absorbs microwave energy. It could cause overheating, thermal decomposition of H_2_O_2_ and even thermal degradation of urea. This might explain rather poor yields of reactions conducted under microwave condition where UHP were used as an oxidant in the solid state (Table 1 and Table 2). A presence of polar solvent could overcome mentioned limitations, e.g., high yields of oxidation with UHP in methanol [19] or 1,4-dioxane-water [20] were obtained in microwave assisted reactions.

H_2_O_2_ encapsulated in silica matrix shown similar activity in the investigated oxidation reactions as 30% H_2_O_2_ (Table 1 and Table 2). However, the composite exhibited lower activity in epoxidation of cyclooctene carried out in TFE (Table 3), an elongation of the reaction time or increasing of the composite amount improves the yield. On the other hand, fluorous alcohols were found to be excellent activators of H_2_O_2_ and a several mechanisms based on formation of hydrogen bonds between fluorine and H-O-O hydrogen atom were proposed to explain the phenomenon of the H_2_O_2_ activation [16,21,22,23,24,25]. Additional, hydrogen bonds found to be responsible for stablility of H_2_O_2_-silica xerogels. FTIR studies and DFT and B3LYP calculations shown that there are complex interactions between H_2_O_2_, H_2_O and silica network because of hydrogen bonds formation [8]. It may be possible that H_2_O_2_ molecules involved in formation of silica complex are not activated by TFE. This might be a reason of the small decrease of the yield of the epoxidation carried out in TFE with silica xerogel–H_2_O_2_ composite with comparison to 30% H_2_O_2_ or UHP.

Tentative mechanisms of oxidation with H_2_O_2_ encapsulated in silica xerogel could be proposed. However, further investigations are needed to prove which of supposed mechanisms (below) taking place.

**Figure 3:**
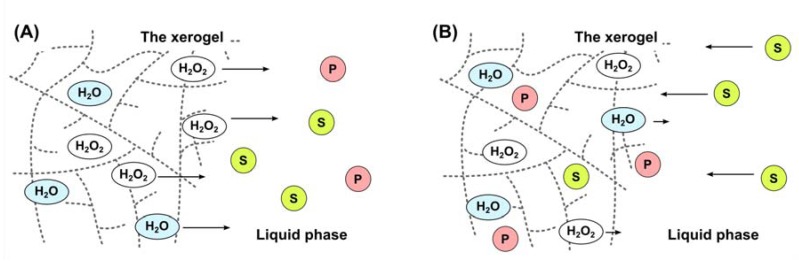
Proposed mechanisms for the xerogel mediated oxidation: S→P. S: substrate, P: oxidation product. (**A**) H_2_O_2_ and H_2_O are fast released from the xerogel to a reaction mixture. (**B**) Small amounts of H_2_O_2_ are slowly released from the xerogel to a reaction mixture.

(1) H_2_O_2_ and H_2_O are fast released from the xerogel to a reaction mixture (Figure 3A). The silica xerogel acts as inert ‘vessel’ for H_2_O_2_ and H_2_O molecules and does not take a part in an oxidation reaction. 

(2) Small amounts of H_2_O_2_ are slowly released from the xerogel to a reaction mixture due to poor solubility of H_2_O_2_ and H_2_O in the solution (Figure 3B). From that reason, the reaction is carried out inside pores of the silica xerogel or on its surface thus additional interactions between the solid support and reagents might occur. 

(3) A combination of the two above mechanisms. H_2_O_2_ and H_2_O is released from the composite to a reaction mixture and the silica xerogel acts both as a solid support for the oxidant and also takes a part in an oxidation reaction, because of a specific interactions silica matrix–reagents.

It seems that the composites may be modified to enhance their properties. It is well-know that silica xerogel dopped with metals could be prepared by drying a mixture of aqueous H_2_O_2_ and silicic acid or silica sol (obtained by e.g., cation exchange method or by hydrolysis of tetraethoxysilane) with a metal salt or a metal sol (e.g., Mg, Al, Ti, Zr, Sn) [26,27]. If the supposed mechanism 2 or 3 is valid, the metallosilica xerogels could be considered as a matrix for H_2_O_2_ encapsulation with potential catalytic sites active in oxidation reactions. Moreover, silica xerogels might be modified by addition of water-soluble polymers forming adducts with H_2_O_2_ such as poly(*N*-vinylpyrrolidinone) [28,29,30] or poly(*N*-vinylcaprolactam) [31,32] to develop tailor-made composites, e.g., with tuned H_2_O_2_ release rate, which is especially important if the hypothetical oxidation mechanism 1 is correct.

## 3. Experimental Section

### 3.1. Equipment

The yield of the reactions was determined by a gas chromatograph coupled with flame ionization detector-GC-FID 6850, Agilent (Santa Clara, CA, USA). Microwave assisted reactions were carried out in a multimode microwave reactor Magnum, Ertec (Wrocław, Poland). FT-IR spectra were obtained by a Biorad STS 165 spectrometer using the KBr pellet technique. FT-Raman spectra were collected by means of an EZRaman-M spectrometer in a range from 250–2340 cm^−1^, using 785 nm excitation diode laser.

### 3.2. Materials

Amberlite IR-120 (H^+^ form), octanal, octanoic acid, 2-octanol, 2-octanone, *cis*-cyclooctene, cyclooctene oxide, 1,1,1-trifluoroethanol (TFE), Na_2_WO_4_·H_2_O, UHP, tetrabutylammonium hydrogen sulfate (TBAHS) and 1,6-dibromohexane were purchased from Sigma-Aldrich and used as received. 30% solution of H_2_O_2_ was from POCH (Gliwice, Poland) and “glass water” R-145 was obtained from Cazet (Łazy, Poland). 

### 3.3. Preparation of Silica Xerogel-Hydrogen Peroxide Composites by the Sol-Gel Method

The composites were prepared according to the literature procedure with some modifications [7]. Silicic acid was obtained from water glass by the cation-exchange method. Water glass (4 g) was diluted with distilled water (16 g). The solution was passed through a bed of cation-exchange resin (Amberlite, 80 g) in a glass column (O.D. 25 mm × 600 mm). The bed was washed with distilled water (40 mL) and effluents were combined and mixed with 30% of H_2_O_2_ (20 mL) without addition of any H_2_O_2_ decomposition inhibitor. The sol was poured onto a Petri dish and dried under hood at room temperature for 48h to give 5–10g of the composite (xerogel). Then the hydrogen peroxide content (%H_2_O_2_) in the composite was determined by iodometric titration and silica content (%SiO_2_) was estimated by drying of the xerogel at 150 °C to a constant weight. Water content was approximated as: %H_2_O = 100-%H_2_O_2_-%SiO_2_, while loading efficiency (LE) was calculated from the equation: *LE*(%) = (*m_x_*/*m_s_*) × 100 where *m_x_* and *m_s_* are weight of H_2_O_2_ in sol and xerogel, respectively [7].

### 3.4. Model Reactions

#### 3.4.1. Octanal Oxidation

Octanal (1.05 g, 10 mmol), TBAHS (0.02 g, 0.06 mmol), 1,6-dibromohexane as an internal standard (100 µL) and the oxidant–equivalent of 12 mmol H_2_O_2_ (30% H_2_O_2_ 1.36 g; UHP 1.13 g; or the xerogel 1.02 g) were mixed in a vial and heated at 90 °C for 2 h. After that, the reaction mixture was diluted by ethyl acetate (3 mL), dried over MgSO_4_ and the solution was and passed through a short pad of MnO_2_ to decompose residue of H_2_O_2_. The solution was analyzed by GC-FID to determining octanoic acid yield. Retention times of the substrate and the product were compared with authentic samples.

#### 3.4.2. 2-Octanol Oxidation

2-Octanol (0.98 g, 7.5 mmol), Na_2_WO_4_∙H_2_O (0.1 g, 0.3 mmol), TBAHS (0.1 g, 0.3 mmol), 1,6-dibromohexane as an internal standard (100 µL) and the oxidant–equivalent of 12 mmol H_2_O_2_ (30% H_2_O_2_ 1.36 g; UHP 1.13 g; or the xerogel 1.02 g) were mixed in a vial and heated at 80 °C for 30 min. After that, the reaction mixture was diluted with ethyl acetate (3 mL), dried over MgSO_4_, and passed through a short pad of MnO_2_ to decompose the residue of H_2_O_2_. The solution was analyzed by GC-FID to determining 2-octanone yield. Retention times of the substrate and the product were compared with authentic samples.

#### 3.4.3. Cyclooctene Epoxidation

*cis*-Cyclooctene (0.45 g, 4 mmol), TFE (3.3 mL), Na_2_WO_4_∙2H_2_O (0,013 g, 0.04 mmol), 1,6-dibromohexane and as internal standard (100 μL) and the oxidant–equivalent of 7 mmol H_2_O_2_ (30% H_2_O_2_ 0.80 g; UHP 0.65 g; or the xerogel 0.60 g) were mixed in a vial and heated at 60 °C for 3h. After that, the reaction mixure was diluted with dichloromethane (2 mL), dried over MgSO_4_ and passed through a short pad of MnO_2_ to decompose the residue of H_2_O_2_. The solution was analyzed by GC-FID to determining cyclooctene oxide yield. Retention times of the substrate and the product were compared with authentic samples.

### 3.5. Microwave Assisted Reactions

Microwave assisted reactions were carried out in the microwave reactor equipped with a magnetic stirrer and upright condenser. Temperature was monitored by a universal fiber-optic sensor (FTI-10, FISO, Canada) immersed directly in a reaction mixture. Reactions time and temperature are indicated in Table 1–Table 3.

## 4. Conclusions

In summary, the results presented in this paper clearly show that silica xerogel–hydrogen peroxide composites obtained by the sol-gel technique are useful oxidizing agents, with an activity similar to 30% H_2_O_2_. Moreover, they have several advantages: they are easy to handle, cheap solids, made from readily available reagents (water glass and 30% H_2_O_2_). Additionally, unlike other solidified H_2_O_2_ forms such as sodium percarbonate or sodium perborate, the composite does not change the pH of a reaction mixture. Silica xerogel–hydrogen peroxide composite exhibits also advantages over UHP. For instance silica (easy to remove by filtration) and H_2_O are the only by-products of the silica based material decomposition. Undesired contamination by urea, its decomposition products or other urea side-reactions can be avoided if the silica xerogel is used. In opposition of UHP complex, H_2_O_2_ is not strongly bonded in the silica matrix thus can be easier released to the reaction solution without heating. Additionally, the xerogel is more easily heated by microwave irradiation than UHP and could be used both as oxidizing agent and as a solid support for microwave assisted solvent free reactions.
